# Creating an "enabling environment" for taking insecticide treated nets to national scale: the Tanzanian experience

**DOI:** 10.1186/1475-2875-4-34

**Published:** 2005-07-22

**Authors:** Stephen M Magesa, Christian Lengeler, Don deSavigny, Jane E Miller, Ritha JA Njau, Karen Kramer, Andrew Kitua, Alex Mwita

**Affiliations:** 1 National Institute for Medical Research, P.O. Box 9653, Dar es Salaam, Tanzania; 2 Swiss Tropical Institute, P.O. Box, 4002 Basel, Switzerland; 3 Population Services International, P.O. Box 33500, Dar es Salaam, Tanzania; 4 WHO Country Office, P.O. Box 9292, Dar es Salaam, Tanzania; 5 National Malaria Control Programme, Ministry of Health, P.O. Box 9083, Dar es Salaam, Tanzania

## Abstract

**Introduction:**

Malaria is the largest cause of health services attendance, hospital admissions and child deaths in Tanzania. At the Abuja Summit in April 2000 Tanzania committed itself to protect 60% of its population at high risk of malaria by 2005. The country is, therefore, determined to ensure that sustainable malaria control using insecticide-treated nets is carried out on a national scale.

**Case description:**

Tanzania has been involved for two decades in the research process for developing insecticide-treated nets as a malaria control tool, from testing insecticides and net types, to assessing their efficacy and effectiveness, and exploring new ways of distribution. Since 2000, the emphasis has changed from a project approach to that of a concerted multi-stakeholder action for taking insecticide-treated nets to national scale (NATNETS). This means creating conditions that make insecticide-treated nets accessible and affordable to all those at risk of malaria in the country. This paper describes Tanzania's experience in (1) creating an enabling environment for insecticide-treated nets scale-up, (2) promoting the development of a commercial sector for insecticide-treated nets, and (3) targeting pregnant women with highly subsidized insecticide-treated nets through a national voucher scheme. As a result, nearly 2 million insecticide-treated nets and 2.2 million re-treatment kits were distributed in 2004.

**Conclusion:**

National upscaling of insecticide-treated nets is possible when the programme is well designed, coordinated and supported by committed stakeholders; the Abuja target of protecting 60% of those at high risk is feasible, even for large endemic countries.

## Background

Like most countries in sub-Saharan Africa, Tanzania carries a heavy malaria disease burden. It is estimated that 28 million citizens are exposed to the risk of stable malaria, resulting in 16 million clinical episodes per year and 100,000 child deaths – over 25% of total deaths [1]. In addition, malaria represents the leading cause of outpatient attendance for children and adults (38% and 32%, respectively) and the cost to health services is, therefore, considerable. The negative impact on the socio-economic development of the country is undoubtedly large. In order to address this enormous burden, Tanzania recently developed a national malaria medium-term strategic plan [1]. With regard to primary prevention, the emphasis has been put on the widespread use of insecticide-treated nets (ITNs). Globally, ITNs have been shown to reduce morbidity and mortality from malaria substantially in over 80 settings [2].

A target of 60% coverage of ITNs by 2005 in the groups bearing the highest malaria risk (young children and pregnant women) was set at the meeting of African Heads of State on malaria in Abuja, Nigeria, in April 2000. This has provided a clear goal for the way forward and stressed the need to rapidly expand access to, and use of, ITNs. Here, a summary of the extensive Tanzanian experience with ITNs, as well and the long and complex process leading to a national scale-up of ITNs, are presented. This paper also attempts to review some of the issues that are crucial for this process, with the hope that this experience might prove useful to other countries.

## Case Description

### ITN developments in Tanzania, 1983 to 2005

Over the last twenty years, much work has taken place on ITNs in Tanzania (Table 1). Various research organisations, donor agencies, non-governmental organizations (NGOs), the private sector and government agencies have been involved in improving this tool and preparing large-scale expansion.

**Table 1:** The critical path of insecticide-treated nets (ITN) research and implementation in Tanzania, 1983 to 2004.

**Critical path of insecticide-treated nets research and Implementation in Tanzania**				
**Efficacy studies**		**Effectiveness studies**	**Policy developments**	**Going to scale**

Reducing malaria vector exposure (including net and insecticide developments)	Reducing malaria morbidity and mortality	Impact (morbidity and mortality) and cost assessment in pilot programmes	National strategies and partnerships for an enabling environment	National ITN strategy and policy NATNETS

1983–1995	1985–1995	1992–2000	1997–2000	>2000

A number of studies have focused on the entomological action of ITNs [3-6]. Following this initial work a number of small-scale trials demonstrated epidemiological impact under controlled conditions – also called efficacy [7-11].

Subsequently, the emphasis was put on larger trials and exploring community-wide benefits of ITNs on both morbidity and mortality. Impact under programme conditions (effectiveness) was demonstrated in 1997–2000 by the Kilombero Net Project (KINET) with a reduction of 27% in the risk of dying associated with ITN use [12]. Even larger beneficial effects were seen for anaemia and parasitaemia in children [13] and in pregnant women [14]. This programme also demonstrated the cost-effectiveness of ITNs under programme conditions [15], with a cost per death averted of USD 1018, making ITNs comparable to childhood immunization.

While studying impact much was also learned about different approaches to ITNs and insecticide distribution, either based on community mechanisms [16-18] or on social marketing [19,20]. Tanzania was also the place for an international ITN meeting in 1994 attempting to bring together all the early experience of ITN implementation, and resulting in the publication of the first book dedicated to this topic [21].

An important development in Tanzania was the design and testing of insecticide home treatment kits [22,23]. Further studies on the issue of net re-treatment highlighted the difficulties associated with sustaining this behaviour [24]. Recently, work was also done on the state of existing polyester nets [25], as well as the remarkable state of Olyset™ long-lasting nets 8 years after their introduction [26].

This large body of work demonstrated that ITNs were highly efficacious, as well as effective, that they represented a cost-effective intervention, and that feasible approaches existed for large-scale expansion. By 2000, two social marketing programmes were operating. Firstly, the Kilombero Net Project (KINET) managed by the Ifakara Health Research and Development Center (IHRDC) and the Swiss Tropical Institute (STI), which has been operating since 1997 in two southern districts [19]. Secondly, the Social Marketing for ITNs project (SMITN) implemented by Population Services International (PSI) which started in 9 districts in 1998 and which went national in mid-2000 (SMITN Phase 2).

From the population census in 2002, the Tanzania population has been estimated at 34,569,232 (mainland 33,584,607) living in 6,996,036 (mainland 6,811,087) households [27]. With a total population at risk of 28 million [28]. and two users on average per net, a minimum standing crop of 14 million treated nets are required to protect the whole population, while 8.4 million nets are required for an overall coverage of 60%. In addition, the same number of treatment kits are required in order to re-treat every net once per year until the introduction of long-lasting insecticidal nets (LLIN). While the official target of NATNETS is to protect 60% of those at high risk of dying from malaria (pregnant women and children under five years), a national programme must obviously also take into account the fact that the rest of the population wants to be protected. Overall ITN distribution targets are hence calculated for the whole population (bearing in mind that only high-risk groups will benefit from a subsidy).

At the 1999 International Conference on ITNs in Dar es Salaam, Tanzania, a call was made to go beyond projects and programmes towards changing norms on a national scale [29]. This ultimately requires working towards a future in which all those in need of protection in malarious areas enjoy easy access to ITNs and use them consistently.

### A concerted national action for ITNs in Tanzania

**Figure 1:**
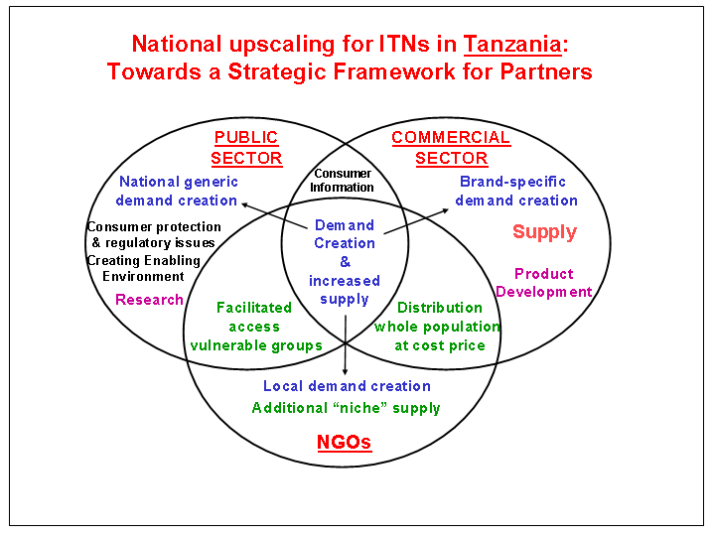
Strategic framework for ITN Scaling up in Tanzania.

During the International ITN Conference in Dar es Salaam in 1999, an opportunity was seized to hold the first ITN stakeholder meeting in the country with a focus on national scaling up. The meeting attracted representatives from the Tanzanian private sector (mosquito nets and insecticide manufacturers, marketing agencies), the public sector (Ministry of Health – MoH), the research and academic communities, NGO's, bilateral donors (UK, Netherlands, Switzerland) and multilateral agencies (UNICEF, WHO). A consensus was reached on the need for a forum including all stakeholders to plan an overall strategy of how Tanzania should proceed. A planning meeting was held in early November 1999 and at this meeting PSI was asked by the MoH to take the lead in organising a large meeting that took place in March 2000. Over 40 stakeholders, representing all major constituencies, attended the workshop. The key outcome was the establishment of a Task Force representing all the stakeholder groups and the framing of roles for all stakeholders (Figure 1). As a first step, this Task Force, under the chairmanship of the MoH, commissioned and facilitated the drafting of a "National Strategic Plan for Insecticide-Treated Nets in Tanzania", which was completed in September 2000, then refined and endorsed by all parties at a second workshop attended by over 50 stakeholders [30]. In a final step, MoH directors officially endorsed the strategic plan in November 2000. The three core concepts of the national ITN strategy (**NATNETS**) are:

(1) Increased demand creation for ITNs.

(2) A national public-private partnership for developing a sustainable domestic commercial ITN market.

(3) Targeted subsidies aimed at high-risk groups.

A close collaboration among the public, private and NGO sectors was advocated around the issue of **demand creation **and increased supply and use of ITNs (Figure 1). The public sector role is focusing on consumer protection, policy and regulatory issues, as well as generic demand creation, in order to create an ITN-enabling environment. The NGO role focuses on more local, grass-root demand creation and support for specific niche supply. The commercial sector role focuses on supply and distribution, product development, and brand-specific demand creation. Underpinning these is the research community, assisting with product development, implementation research, market research, and monitoring and evaluation. Bilateral donors provide strategic funding support, as well as strategic thinking across sectors.

From 1999 to 2002, the MoH Task Force and its membership of stakeholders have facilitated and coordinated all ITN activities in the country. Key landmarks in this process were (1) a national ITN implementation plan and budget produced by the National Malaria Control Programme (NMCP) and (2) a successful application to the Global Fund to Fight AIDS, TB and Malaria in 2002 to support a targeted national ITN subsidy programme to increase coverage and equity (see below). So far, all stakeholders, including NGOs and multilateral partners have aimed towards coordinating all activities within NATNETS and no alternative programmes have been implemented in the country.

**Figure 2:**
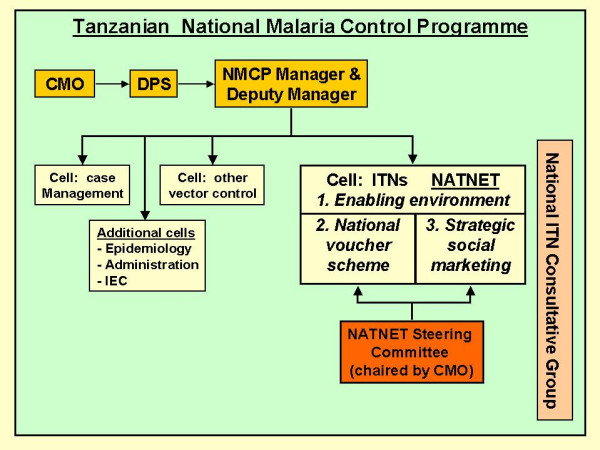
The National Malaria Control Programme structure showing the position of the ITN cell and the main components of the national ITN strategy (NATNETS). CMO = Chief Medical Officer, DPS = Director of Preventive Services, NMCP = National Malaria Control Programme, IEC = Information, Education, Communication.

With a new ITN landscape emerging in the country, a **Steering Committee**, made up of Tanzanian Government departments, PSI, STI and key donors, was created in July 2002 as an overseeing body for NATNETS, under the chairmanship of the Chief Medical Officer (CMO). A second group with a wider membership was created to provide a more general stakeholder forum for all aspects related to the scaling-up process – the **ITN consultative group**. An **ITN cell **was also created within the NMCP in May 2003 to become the executive body coordinating and supporting the ITN process in the country (Figure 2). To this effect, a full time NATNETS Team Leader was recruited in May 2003, together with two professional officers. Support was provided by the MoH and the Swiss Agency for Development and Cooperation (SDC) through the Swiss Topical Institute.

Practically, NATNETS is now based on **three major operational components**:

(1) The ITN cell – a coordination unit to create an enabling environment for taking ITNs to national scale;

(2) Strategic social marketing (SMARTNET) to massively upscale the supply of ITNs;

(3) The Tanzanian national ITN voucher scheme (TNVS) to selectively target pregnant women with highly subsidized nets.

The areas of responsibility of the key ITN stakeholders in the country are presented in Table 2, as an extension from Figure 1. Since these operational components are the basis of NATNET they will be reviewed in turn below.

**Table 2:** Shared roles within the national ITN initiative (NATNETS).

**NATNETS National Malaria Control Programme (NMCP)**	
**ITN cell within NMCP** • Overall coordination of ITN activities, including linkage with NGOs and commercial sector • Advocacy (political, administrative) • Reduction of taxes and tariffs • Legislation on insecticides & consumer protection • Quality control (nets and treatment) • Technical support to districts • Brokering agreements between public sector and commercial sector • Resource mobilisation for other aspects of ITN Strategy • Increasing role in national/local level demand creation • Monitoring and evaluation • **Design & implementation of national voucher scheme**	**Strategic social marketing (SMARTNET)** • Market research and creation of a national ITN logo • Opening new markets in areas not yet reached by commercial markets and attracting commercial distribution into such areas • Marketing support to national net manufacturers/distributors/retailers • Distribution support to national net manufacturers/distributors/retailers • National/local demand creation • Net treatment kit promotion and distribution • Close collaboration with ITN Cell

**Additional key ITN stakeholders**	

**Research community** • Monitoring and evaluation • Implementation research • Market research • Product development	**Commercial sector** • Net production • Net distribution • Branded advertisement • Product development • Market research

### Creating an enabling environment for ITNs

Neither the public sector nor the commercial sector alone can achieve the level of coverage required to meet and exceed the Abuja target. This is evidenced by the fact that the Government of Tanzania and its donor partners spend only USD 5.10 per capita per year on the entire health system [31]. Other partners including NGOs and the private sector spend an additional USD 0.80 per capita. Households, for their part, contribute the largest share of health expenditure, averaging USD 5.35 per capita. Within this limited budget, it would be impossible to allocate a substantial part of public spending to a single intervention for a single disease. Furthermore, the current health structures would be unduly stretched by the distribution of a large volume of valuable goods. There is ample evidence to suggest that nets are a highly attractive commodity once available within a reasonable distance, and at a low price [18,20,21].

As a result, there is a strong consensus both in Tanzania and internationally [32] that the best way forward is a well-coordinated partnership among all ITN stakeholders based on increased demand and supply, a vibrant commercial sector, and a targeted subsidy scheme for those most at risk. In order to create and sustain this partnership a shared vision is required, as well as a number of **enabling factors**. Some of the key enabling factors are: 1) removal of any form of taxation; 2) favourable insecticide regulatory conditions; 3) net quality control issues; 4) generic demand creation by the public sector; and 5) equity of access.

#### Taxes and Tariffs

Affordability of mosquito nets is the major single factor limiting wide and equitable coverage of mosquito nets, especially in rural settings. Removing all taxes and tariffs on nets, netting and insecticide is a relatively simple and very effective way to make ITNs more affordable. In 1995 imported nets and locally manufactured nets were subject to customs duty (tariff) and sales tax, (later value added tax – VAT). In addition, there were licensing requirements (including application and permit fees) and foreign exchange controls limiting imports.

National manufacturing companies experienced a difficult period in the sales of ready-made nets between 1980 and 1992 when the government levied a sales tax of 125%. At this point netting material was not taxed and the sole domestic manufacturer at that time (Sun Flag Ltd) found it more worthwhile to sell netting material to local tailors than to turn netting into nets. As a result, ready-made nets were luxury items for the majority of Tanzanians. In 1994, when ITNs were beginning to be recognized as important tools for malaria control, the sales tax was removed on ready-made nets. This resulted in a substantial price drop and the net manufacturing company began to experience a rise in sales. As a result, a second domestic textile manufacturer started production in 1998 (A-Z Textiles Ltd).

In May 1998, the MoH, with support from PSI and the Programme for Applied Technology (PATH), Canada, convened a meeting in Tanzania with the purpose of promoting the use of treated nets within the country with an emphasis on partnership between the private and public sectors. It brought together representatives of the MoH, Ministry of Finance, Ministry of Trade & Industries, textile manufacturers, insecticide manufacturers, NGO's, bilateral and multilateral organizations. The meeting unanimously agreed that VAT, which was to be introduced for the first time in Tanzania, was going to affect negatively the pricing of nets. It was decided that each stakeholder should petition against the proposed VAT on nets. The meeting document was circulated to the Permanent Secretaries of the Ministries of Health, Finance, Industries & Trade, to the World Bank, WHO and the media. The NMCP was urged to present the national ITN strategy to the MoH so that the Minister could present it at the next Parliamentary session.

Despite these efforts, VAT was introduced on nets and netting materials during the 1998/99 budget. But shortly after, thanks to the stakeholders' intensive lobbying, an amendment was issued by the Ministry of Finance waiving VAT on mosquito nets. Importers of ready-made nets had to pay only 5% import duty. Unfortunately, local producers still had to pay 10% import duty and 20% VAT on raw materials (polyester yarn, thread and packaging materials), utilities and machinery. This put them at a disadvantage compared to imported nets. Further lobbying was, therefore, undertaken. This finally resulted in the 1999/2000 Finance Bill that declared mosquito nets and insecticides "zero rated" items. The bill classified ready-made mosquito nets as essential drugs, like other pharmaceuticals, which attract only 5% import duty and are exempted from VAT. However, in revised legislation taking effect in July 2002, these provisions were again removed. In response to this, the national ITN Task Force had further consultations with government authorities including the Tanzania Revenue Authority. From the end of 2004, all netting items were again zero-rated for VAT and the VAT on imports of inputs (mainly the yarn) can be re-claimed against proof that the material was used for netting manufacture.

This experience shows clearly that success depends on continuously lobbying the finance and tax departments, arguing that the economic and health benefits of ITNs outweigh concerns about loss of government tax revenue. Tanzania's experience also illustrates the need for vigilance by the health authorities and ITN stakeholders since taxes and tariffs are constantly shifting. As a result of these efforts the local manufacturing of nets has been stimulated and the average retail price of Tanzanian nets has gone down from over USD 5 in 1995 to less than USD 3.5 in 2004. This has made ITNs substantially more affordable. With better market prospects, a third net manufacturer started selling nets in 1999 (TMTL Ltd), bringing the combined annual net production to over 5 million. Recently, a fourth manufacturer (Motex, Moshi Textile Mills, Ltd.) has started production. Taxes and tariffs on insecticide are also a potential issue, but so far all insecticide used for ITNs has been imported by tax-exempted projects and this issue has not arisen. It will, however, have to be dealt with in the future.

#### Regulatory issues concerning insecticides

Regulatory issues are important in a number of aspects including insecticide registration, the definition of authorized retail outlets, consumer rights and product quality. Efforts have been made to speed up the process of insecticide registration by proposing to fast-track those products already approved by the WHO Pesticides Evaluation Scheme (WHOPES).

Another issue was that previously only registered pharmacies were allowed to sell home insecticide re-treatment kits. After a number of meetings and a thorough review, the Tanzanian Pesticides Research Institute (TPRI) as well as the MoH agreed that such kits could be distributed more widely by the SMITN and KINET social marketing projects. The TPRI regulations had to be amended to this effect and thanks to prompt action by all concerned this was agreed in 2000. In a further step and following a private-public sector agreement in 2002, insecticide kits were bundled with all nets leaving Tanzanian factory doors for the domestic market. Hence, from the end of 2002 onwards, the vast majority of nets are sold with insecticide in the country (some unbundled, imported, poor quality Far-East Asian nets are sometimes found). SMARTNET research showed that 92% of all nets sold with an insecticide kit were treated.

### Strategic social marketing

In 2002, the PSI ITN social marketing project (SMITN) took on a new role under the name "Strategic Social Marketing for Expanding the Commercial Market for ITNs in Tanzania" (SMARTNET). SMARTNET is a 5-year initiative managed by PSI-Tanzania and funded by the British and Netherlands development aid programmes. Under SMARTNET the role of social marketing evolved substantially compared to previous projects. SMARTNET's primary focus has moved away from promoting and distributing PSI over-branded ITNs, to assisting the Tanzanian net manufacturers to expand their wholesale and retail network in the country, especially in rural areas. It is expected that this will lead to a sustainable and vigorous net market by 2007. Support includes nation-wide multi- and single-brand advertisement of the net manufacturers' products, identification of primary agents/wholesalers/retailers, transport subsidies to remote locations and general support. All companies benefit basically from the same support, although in the end this support is obviously dependent on how active they become in developing the market. Multi-branded advertisement is achieved by including the logos of all manufacturers on the promotional materials developed by SMARTNET. An early success of SMARTNET has been to convince the Tanzanian net manufacturers to bundle all their nets destined for the Tanzanian market with insecticide treatment kits. This was made possible by supplying kits to them very cheaply (USD 0.15) and by making sure that all the manufacturers participated, hence avoiding unfair competition from untreated nets.

In 2004 the combined sales of Tanzanian manufacturers on the domestic market had reached nearly two million insecticide-bundled nets and the projections for 2005 are over 2.5 million nets as a result of the start of the Tanzanian National Voucher Scheme (TNVS). This illustrates clearly the growth potential of the market when assisted strategically by the public sector.

In addition, SMARTNET is continuing to develop a national distribution network for insecticide treatment kits (branded *Ngao*^® ^*and Ngao ya Maji*^®^), since selling insecticide kits does not constitute an attractive long-term commercial market due to the development of long-lasting insecticidal nets (LLIN). The first of these, the Olyset^® ^net developed by Sumitomo Corp. is now being manufactured by A-Z Textiles Ltd in Tanzania and will soon be available on the local market. SMARTNET intends to promote Olyset^® ^nets as soon as they become available. In 2004, nearly 2.2 million additional insecticide treatment kits were sold.

By developing the supply chain, SMARTNET has been crucial to the success of the TNVS, which cannot work unless ITNs are available everywhere in the country. On the other hand, the TNVS helps develop and sustain the ITN market by generating a large demand for these products, especially among the poorer segments of the population (see below).

### The Tanzanian National Voucher Scheme (TNVS)

The Tanzania National Voucher Scheme (TNVS) is a five-year scheme supported by the Global Fund to Fight AIDS, Tuberculosis and Malaria [33], which started operating in October 2004, after an 18 months preparation phase. The TNVS is modelled on a similar voucher scheme implemented by the KINET project from 1997 to 2001 [33]. It aims at giving every pregnant woman attending an antenatal clinic a printed voucher with a face value of TShs 2750 (USD 2.75 in 2004). This voucher can be used to purchase an ITN from any participating commercial retailer at a discounted price. The pregnant woman has to spend between USD 0.5 and USD 1.5 to get her ITN (current retail prices: USD 3 to 4, according to shape, colour and size). The retailers then redeem the voucher with their wholesaler and the wholesaler with regional teams working for the programme or with the net manufacturers. In addition, a free insecticide treatment kit is given to all women attending antenatal care, as well as to mothers attending vaccination clinics with their child at 3 and 9 months. Through this additional mechanism infants will be protected by a treated net from before birth until after the first year of life. The main aims of the TNVS is to provide a facilitated and equitable access to ITNs to those groups most at risk of the severe consequences of malaria, i.e. pregnant women (1.4 million every year) and their newborn children.

This approach was chosen instead of direct distribution of free nets for two main reasons: 1) avoiding the complex logistical problems of distributing ITNs through the already over-burdened public health system; 2) providing a subsidy mechanism that strengthens the development of a sustainable commercial distribution, especially in rural areas; 3) rapidly addressing equity concerns raised by a purely commercial market; 4) increasing the coverage of antenatal and MCH care.

By February 2005, preliminary results from the 7 initial regions where the TNVS was operating suggested:

• a major increase in the number of retail outlets selling ITNs: 700 retail outlets in 7 regions were involved in the TNVS, of which over 70% were new to the ITN business

• a major increase of sales figures at retail and wholesale level (50 – 80%)

• approximately 80% of the pregnant women in the TNVS areas using the voucher to buy an ITN

• attendance of ante-natal clinics by pregnant women earlier in their pregnancy. As a result of the TNVS clinics have indicated that they see many more women in their first trimester, making it possible to provide better overall health care

• active district involvement in ITN-upscaling activities as a result of intensive advocacy and negotiations with the different ministries involved

Hence, the TNVS is expected to support a rapid expansion of ITN use by both pregnant women and infants. Moreover, the TNVS is expected to support and encourage private sector involvement in the manufacture of ITNs and their delivery to poor rural communities, since there will be widespread and predictable demand for ITNs by pregnant women. The scheme is coordinated by the national ITN cell and implemented by tendered contractors. This was thought to be the most efficient and effective way of introducing a complex new public health service, and it could provide a model for future developments in the frame of health sector reforms. The ITN Steering Committee is overseeing the TNVS and there is a close coordination with the SMARTNET programme.

### Costing NATNETS

Cost data for NATNETS are currently being collected systematically in the frame of routine evaluation and monitoring activities. Following cost figures are currently available.

The annual cost of the ITN cell amount to USD 364,000. This amount includes staff, management, running cost and technical support. No data are currently available on the cost of the SMARTNET component. Finally, the annual cost of the TNVS will amount to USD 5,385,000 once the programme operates at national level (1.2 million vouchers given out per year). Of this amount, USD 3,308,000 (61%) represent the value of the vouchers and the insecticide re-treatment kits, USD 1,384,000 (26%) will be the cost of logistics (distribution of vouchers and their redemption), and USD 693,000 (13%) will be used for training and promotion. Hence, the cost per net given out using a voucher amounts to USD 4.49, to which the recipient will have to add USD 0.5 to USD 1 to purchase an ITN.

## Discussion and Lessons Learned

While this paper attempts to present the Tanzanian experience as objectively as possible, it is written by individuals who were all involved in these developments. In order to ensure an independent assessment of NATNETS, an evaluation and monitoring programme (including costing) is currently carried out by a joint team from the Ifakara Health Research and Development Center and the London School of Hygiene and Tropical Medicine. The evaluation does not entail measuring the impact of NATNETS on morbidity and mortality from malaria, since this has been demonstrated beyond doubt in many settings, including in Tanzania [2,12,13]. In any case, regular monitoring of child survival parameters will be available, either through existing demographic surveillance systems (of which there are currently four in the country) or through the regular Demographic and Health Surveys.

The strong development of the commercial sector for ITNs since 1995 is testimony to the success of NATNETS. So far, a number of key lessons have been learned from 1983 to 2004.

### A coordinated Public-Private Alliance

Tanzania has been special in having not only a large number of ITN activities, but also a substantial group of motivated ITN stakeholders. From early on this group has acted in a concerted way through the national ITN Task Force and integrated all ITN activities in the country. As a result, many initiatives could be launched and coordinated to allow a true national scale-up to be considered from 2002 onwards. The role of the MoH has always been very supportive, especially in promoting an enabling environment. Politicians have been targeted regularly and have clearly supported this process. Finally, bilateral and multilateral donors have been generously supporting this process, providing a good model for the funding of large-scale malaria control initiatives. This public-private partnership could serve as a wider example in the health sector, nationally as well as internationally.

### Coverage and equity

SMITN and KINET data suggest that affordability remains a significant obstacle to net use, especially for the poorest. Recent data confirm a socio-economically stratified gradient in treated and untreated net ownership and re-treatment rates [35,36], although this gap is narrowing over time. As household coverage rates increase overall, and with the beneficial effects of the TNVS, coverage should rapidly increase in the lower socio-economic quintiles and harder-to-reach rural areas.

The data in Table 3 show coverage figures from a household survey undertaken by NMCP in 14 districts in Tanzania in 2001 and 2003, prior to the TNVS (unpublished data). The rapid growth in coverage in children and pregnant women is apparent. It is certain that the 2004 national coverage figures will be substantially higher, given current ITN sales figures and the launch of the TNVS. A further NMCP household survey is planned for 2005.

**Table 3:** ITN coverage in 14 districts in Tanzania, 2001 and 2003. Source: National Malaria Control Programme. ITN = insecticide-treated net.

Target group	Percentage sleeping under an ITN		Percentage sleeping under an untreated net		Total (ITN and untreated)	
	
	2001	2003	2001	2003	2001	2003
Children under 5	15	26	31	27	41	58
Pregnant women	8	21	28	21	29	49

### Net manufacturers

Tanzania is in a unique situation in sub-Saharan Africa by having no less than four domestic mosquito net manufacturers with a total annual net production of over 5 million nets, of which nearly 2 million entered the domestic market in 2004. The Tanzanian manufacturers have made initial progress in expanding the distribution network for nets, but were unlikely to expand further to under-served rural areas on their own. This situation has called for specific strategies to support this development, leading to the launching of the SMARTNET and TNVS programmes.

Removing all taxes on nets and netting did lead to a substantial decrease in retail prices. As a result of lower prices, demand for nets increased dramatically and this allowed the manufacturers to develop the market. This lesson might be of use to other endemic countries which have not yet implemented ITN tax removal, one of the commitments from the Abuja Summit.

### Treated versus untreated nets

The benefits of using a treated net compared to an untreated net are not universally understood and valued by consumers. Up to 2002, all ITN projects held jointly less than 30% of the market share, while the remaining 70% were untreated nets sold through commercial channels. Untreated nets have a clear competitive advantage because of the extreme price sensitivity of mosquito control products. Unfortunately, untreated nets provide less than half the public health impact of treated nets [2] and a significant effort is required to ensure that only treated nets are sold. An important step has been made in this direction with the 100% bundling policy agreed by all major net manufacturers in the country and the readiness to engage in the production of LLIN.

### Main outstanding challenges

The most obvious current challenge is to make the TNVS operate smoothly at national scale, with operational stability and limited fraud.

Funding for the ITN cell is secured until 2008, that for SMARTNET until June 2007 and that for the TNVS until end 2007. Long-term financing needs to be secured for all components, both from external sources and from the existing MoH resources.

A second challenge will be to bring LLIN as quickly as possible onto the market. Currently, one brand of LLIN is available on the Tanzanian market but its production capacity is insufficient and its retail price is too high to make it widely available (over USD 7). Attention is currently devoted to secure access of the LLIN technology to the Tanzanian manufacturers of polyester nets.

## Conclusion

Tanzania has been among the leading African countries in the development and promotion of ITNs. The Ministry of Health has realised and appreciated the role of ITNs in the control of malaria. The country enjoys a unique situation by having effectively stimulated a strong local net industry and distribution network. There is also a committed team of ITN stakeholders from the public and private sectors who have pioneered the scaling up process in a coordinated way, in the spirit of the Roll Back Malaria Partnership. As a result, different components have been implemented to complement each other. It is expected that the NATNETS initiative will enable Tanzania to become the first large African country to meet the Abuja target for ITN use, and hence reduce substantially and sustainably the morbidity and mortality due to malaria over the coming years.

## List of Abbreviations

CMO Chief Medical Officer

DPS Director of Preventive Services

IEC Information, Education, Communication

IHRDC Ifakara Health Research and Development Center

ITNs Insecticide-Treated Nets

KINET Kilombero Net Project

LLIN Long-Lasting Insecticidal Net

MCH Mother and Child Health

MoH Ministry of Health

NATNETS National ITN Strategy

NGO Non-Governmental Organization

NMCP National Malaria Control Programme

PATH Programme for Applied Technology

PSI Population Services International

RBM Roll Back Malaria

SMARTNET Strategic Social Marketing for Expanding the Commercial Market

for ITNs in Tanzania

SMITN Social Marketing for ITNs Project

STI Swiss Tropical Institute

TNVS Tanzania National Voucher Scheme

TPRI Tanzania Pesticides Research Institute

USD United States Dollar

VAT Value-Added Tax

WHO World Health Organization

WHOPES World Health Organization Pesticides Evaluation Scheme

## Authors' contributions

Stephen M. Magesa, conceived the idea of writing this publication, was involved in the design, providing intellectual content and the main writing of the manuscript. Christian Lengeler was involved in the design, providing intellectual content and substantial writing. Don deSavigny was involved in the design, providing intellectual content and writing. Jane E. Miller was involved in the design, providing intellectual and factual content and a critical review of the manuscript. Ritha J.A. Njau, was part of the team that conceived the idea of writing this publication, was involved in the design, providing intellectual content and a critical review of the manuscript. Karen Kramer provided some essential factual inputs and reviewed critically the manuscript. Andrew Y. Kitua, conceived the idea of writing this publication, was involved in the design, providing intellectual content and a critical review of the manuscript. Alex Mwita, provided intellectual and factual content and critically reviewed the manuscript
